# COVID-19 Vaccine Perceptions, Intentions, and Uptake Among Young Adults in the United States: Prospective College-Based Cohort Study

**DOI:** 10.2196/33739

**Published:** 2021-12-15

**Authors:** Stephen Gurley, Brady Bennett, Patrick Sean Sullivan, Maryellen Kiley, Jamie Linde, David Szczerbacki, Jodie Guest

**Affiliations:** 1 School of Medicine Emory University Atlanta, GA United States; 2 Rollins School of Public Health Emory University Atlanta, GA United States; 3 Curry College Milton, MA United States

**Keywords:** COVID-19, vaccine, hesitancy, college, higher education, race, perception, intention, uptake, prospective, cohort, demographic, minority, young adult

## Abstract

**Background:**

Uptake of the COVID-19 vaccine among US young adults, particularly those that belong to racial and ethnic minorities, remains low compared to their older peers. Understanding vaccine perceptions and their influence on vaccination uptake among this population remains crucial to achieving population herd immunity.

**Objective:**

We sought to study perceptions of COVID-19 vaccines as well as intended and actual vaccine uptake among one population of college students, faculty, and staff.

**Methods:**

As part of a larger study aimed at investigating the dynamics of COVID-19 transmission, serology, and perception on a college campus, participants were asked about their views on the COVID-19 vaccine in February 2021. Vaccination status was assessed by self-report in April 2021. Logistic regression was used to calculate prevalence ratios with marginal standardization.

**Results:**

We found that non-White participants were 25% less likely to report COVID-19 vaccination compared to White participants. Among those who were unvaccinated, Black and other non-White participants were significantly more likely to indicate they were unwilling to receive a COVID-19 vaccine compared to White participants. The most common reason for unwillingness to receive the vaccine was belief that the vaccine approval process was rushed.

**Conclusions:**

There are racial differences in perceptions of the COVID-19 vaccine among young adults, and these differences might differentially impact vaccine uptake among young racial and ethnic minorities. Efforts to increase vaccine uptake among college populations might require campaigns specifically tailored to these minority groups.

## Introduction

Since its first reported case in the United States in January 2020, the ongoing COVID-19 pandemic has caused over 4 million deaths globally, and has severely disrupted global economies, educational and workplace practices, social gatherings, and daily behaviors [1]. To combat the spread of SARS-CoV-2, the virus that causes COVID-19, numerous vaccines targeting components of the virus have been rapidly developed. Since February 2021, three vaccines against SARS-CoV-2 have been granted Emergency Use Authorization (EUA) by the US Food and Drug Administration: BNT162b2 (Pfizer-BioNTech), mRNA-1273 (Moderna), and Ad26.COV2.S (Janssen/Johnson & Johnson) [2]. Although there is wide variability in vaccination rates among and within states, over 330 million total doses have been administered nationwide as of July 7, 2021, with 55.1% of adults having received one or more doses [3].

To support programs to increase vaccination, greater attention is now being paid to community perceptions of these vaccines and how they relate to vaccine uptake and hesitancy, particularly among racial and ethnic minorities [4]. Several studies have found sociodemographic differences in vaccine hesitancy across the population, with significantly higher rates of vaccine hesitancy among those who are non-White compared to Whites and those without a 4-year advanced degree compared to those with a 4-year advanced degree [5]. Many attribute this concentration of hesitancy among non-White groups, particularly among Black Americans, to centuries of structural and medical racism that have resulted in unequal treatment of minority populations in the American health care system [6]. Understanding the unequal distribution of COVID-19 vaccine uptake and hesitancy is important as vaccination campaigns across the United States aim to reach sufficient community coverage to surpass the herd immunity threshold, estimated to be between 80%-90% for COVID-19 [7].

Young adults, including college or university students, are more likely to be asymptomatic or paucisymptomatic carriers of SARS-CoV-2 compared to their older counterparts [8]. American college students also frequently live together with other students in congregate dormitory settings or off-campus housing, which has led to numerous outbreaks of COVID-19 among young adults on college campuses [9-11]. These outbreaks have had spillover effects into neighboring communities and act as super-spreader–like events [12]. Despite this increased potential for viral spread, young adults are among the least likely age group to receive vaccines against other respiratory pathogens, such as for seasonal influenza strains [13]. Seasonal influenza vaccination uptake, which has been suggested as a possible predictor of COVID-19 vaccination uptake [14], has historically been low on college campuses, estimated to be between 8%-39% based on a survey of college students [15] conducted at a time when many schools did not require seasonal influenza vaccination. Current national data reveal that 18- to 29-year-olds are the least likely age group to be vaccinated for COVID-19 [3]. Because of this, it is vital to understand perceptions of the COVID-19 vaccines and predictors of vaccine uptake and willingness among college student communities, overall and stratified by racial and ethnic minority groups. Understanding these patterns of uptake and willingness among college students will help public health officials and student health programs to better promote vaccination opportunities to increase the uptake of vaccines for young adults and college-aged persons.

In this study, we describe results of behavioral and perception surveys administered as part of a longitudinal cohort study of students, faculty, and staff at a small liberal arts college located in Milton, Massachusetts, a suburb of Boston. This college has approximately 2000 undergraduate students, about 1000 of which live on campus in congregate dormitory settings, as well as 300 faculty and 400 staff members. Data were collected at two time points during the Spring 2021 semester: in the first week students returned to campus in early February, and immediately prior to final exams in late April 2021. We report the perceptions and intentions of this population regarding the COVID-19 vaccine as well as vaccine uptake.

## Methods

All students, faculty, and staff members of Curry College electronically received an invitation to learn more about the study and enroll via a HIPAA-compliant, online survey-hosting platform. Interested individuals completed the informed consent process online in either text or video format; we have previously demonstrated that video consent is associated with higher comprehension of consent elements [16]. Consent to participate was documented online. Eligibility criteria were as follows: aged ≥18 years, ability to read and understand English without assistance, being a member of the Curry College residential community during the Spring 2021 semester (February-May 2021); completing at least part of their instruction or job in-person on the Curry College campus in Milton, Massachusetts; intention to remain in the vicinity of Curry College for the entire study period, willingness to comply with Curry College COVID-19 weekly screening requirements, willingness to answer biweekly study surveys electronically sent via email, willingness to participant in venipuncture for blood sample collection, and having no bleeding disorder preventing the use of venipuncture. Eligible participants who did not complete consent before returning to campus were offered enrollment on-site during their first week of classes.

Enrolled participants completed an online baseline survey. The survey collected demographic and socioeconomic information, as well as perceptions regarding COVID-19 risk and the COVID-19 vaccines, vaccine hesitancy, and self-reported vaccine uptake, with individual questions adapted from a validated, national probability-sampled survey used in a larger study of SARS-CoV-2 seroprevalence [17]. Vaccine hesitancy and concerns about the vaccine were assessed with a single item asking about willingness to receive the vaccine when eligible. At the time of the baseline survey in February 2021, most states were only offering the vaccine to those aged >65 years, health care workers, and essential workers. By the time of the final survey in late April 2021, all US adults were eligible for the COVID-19 vaccine. Participants were also asked about their perception of the safety and efficacy of the vaccines and perceptions about the timing of the EUA approval for the vaccines. At the end of the Spring 2021 semester in late April, participants completed a final survey including the same questions about perceptions about COVID-19 vaccines and receipt of vaccination.

Participant status as a health care worker or student (HCW) was established after identifying the primary major or department with which the student, faculty, or staff member was primarily affiliated via school records. Students were categorized as HCWs if their major course of study was either nursing or exploratory health professions. Faculty and staff were categorized as HCWs if their primary department was either the Department of Nursing or the Student Health Center.

Selection fractions and ratios will be calculated for students as a whole as well as for on-campus residential and off-campus commuter students and to assess for possible selection bias among these student subpopulations.

All statistical analyses were conducted with SAS (version 9.4; SAS Institute). Descriptive statistics and frequencies among the study sample results were obtained for all categorical demographic, exposure, and outcome variables in the survey. Fisher exact test was used to determine if there were statistical differences in attitudes toward and willingness to receive the COVID-19 vaccine between several sociodemographic groups, including racial and ethnic groups. To determine whether there were statistical differences in sociodemographic variables and perception of/intention to receive vaccine between those who were vaccinated and unvaccinated in April, predicted prevalence ratios with marginal standardization and confidence intervals were calculated using logistic regression [18]. We also used Fisher exact test to determine if there were statistically significant differences in self-reported vaccine uptake at the final time point between those who reported at baseline that they were unwilling to receive the COVID-19 vaccine and those who reported they were willing.

This longitudinal cohort study was conducted with the approval of the Emory University (STUDY00002096) and Curry College Institutional Review Boards in accordance with all applicable regulations.

## Results

Between February 14-19, 2021, a total of 454 participants enrolled in the study and completed the baseline survey and venipuncture. Of these, 328 (72.8%) completed the second venipuncture and final survey in late April 2021. Most participants were female (333/454, 70.9%), White (366/433, 71.3%), non-Hispanic/Latinx (402/453, 69.2%), and students (308/450, 60.4%; Table 1). The median age of participants was 21 years. Most participants were not HCWs (313/454, 68.9%), as classified by department affiliation and student major (Table 1). In total, about 14% (308/2200) of the total student body participated in the study ulti(Table S1 in Multimedia Appendix 1).

**Table 1:** Demographic characteristics and self-reported COVID-19 vaccination status among students, faculty, and staff at Curry College, Milton, Massachusetts, February 2021.

Demographics			All participants (N=454), n		Characteristic by self-reported vaccination status			
					Unvaccinated (N=149), n (%)^a^		Vaccinated (N=305), n (%)^a^	Prevalence ratio (95% CI)^b^
**Natal sex**								
	Male	121		52 (43)		69 (57)		Reference
	Female	333		90 (27)		243 (73)		1.11 (0.94-1.30)
**Race**								
	White	366		105 (29)		261 (71)		Reference
	Black	33		19 (58)		14 (42)		0.79 (0.63-0.98)^c^
	Other	34		17 (50)		17 (50)		0.79 (0.63-0.98)
**Ethnicity**								
	Non-Hispanic/Latinx	402		124 (31)		278 (69)		Reference
	Hispanic/Latinx	51		25 (49)		26 (51)		0.94 (0.75-1.19)
**Affiliation**								
	Student	308		122 (40)		186 (60)		Reference
	Staff	86		17 (20)		69 (80)		1.45 (1.28-1.64)^c^
	Faculty	56		8 (14)		48 (86)		1.45 (1.28-1.64)
**Work or study in health care setting**								
	Non–health care workers	313		119 (38)		194 (62)		Reference
	Health care workers	141		30 (21)		111 (79)		1.35 (1.18-1.54)^c^

^a^Unless otherwise stated, percentages shown are row percentages.

^b^Prevalence ratios with marginal standardization with 95% CI are from multivariate modified logistic regression models testing associations between predictors and vaccination status.

^c^*P*<.05.

**Figure 1:**
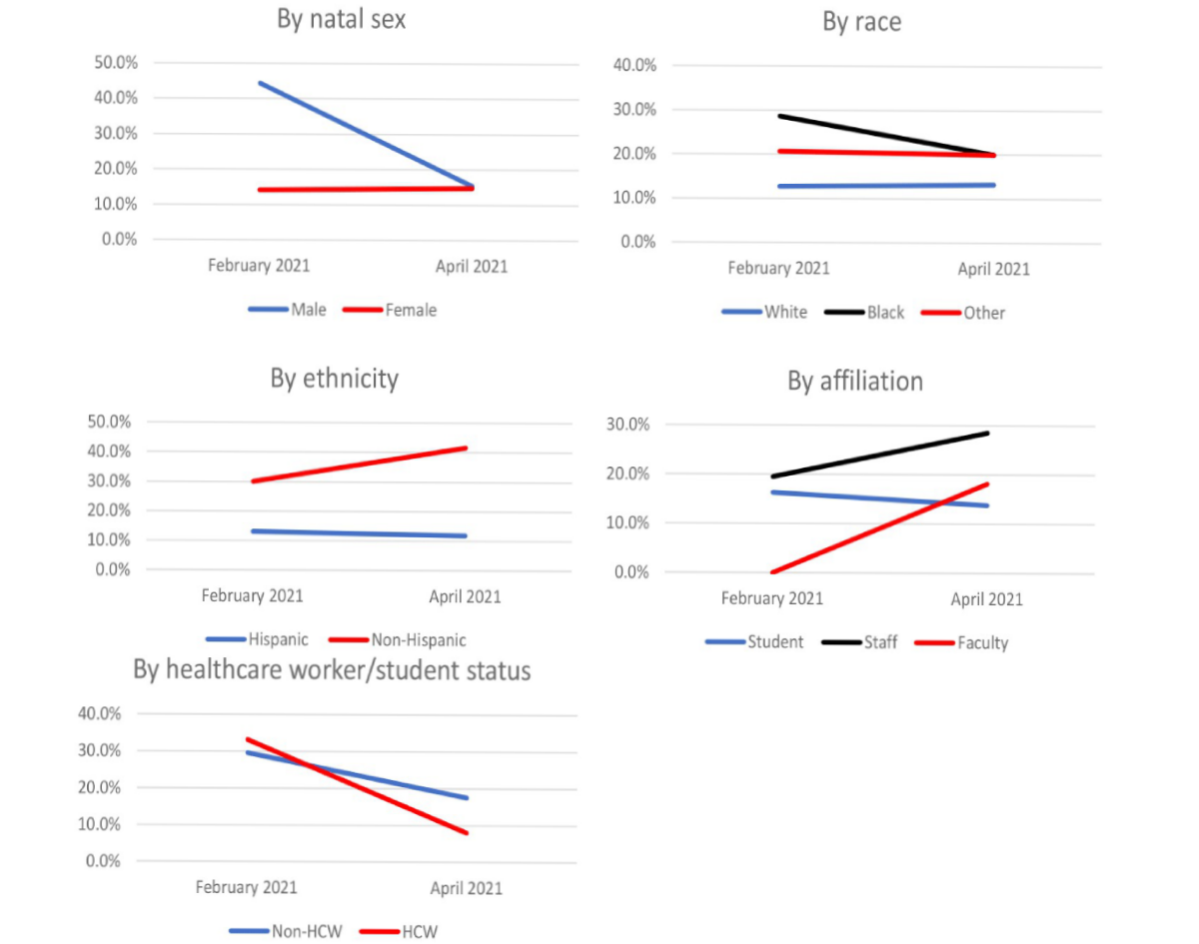
Percentage of participants who self-reported they are unlikely to receive a COVID-19 vaccine. Only those who were unvaccinated at each time point were asked this question (N=341 for time point 1 and N=105 for time point 2). HCW: health care worker.

Participants were asked about their willingness to receive a COVID-19 vaccine and their attitudes regarding COVID-19 vaccines at the baseline time point in February and at the final study survey in April 2021. At baseline, over two-thirds of those who reported they were unvaccinated indicated that they were willing or very willing to receive the COVID-19 vaccine once they became eligible (Figure 1, Table S2 in Multimedia Appendix 1). In comparison, in the final survey, among the 105 participants who reported not having received a dose of a COVID-19 vaccine, nearly 65% reported that they were willing or very willing to get vaccinated once they were able (Figure 1, Table S3 in Multimedia Appendix 1). All Massachusetts residents ≥16 years old have been eligible to receive the COVID-19 vaccine since April 19, 2021. However, in many areas, demand for vaccines exceeded supply, leading to delays in obtaining appointments [19]. Attitudes regarding the COVID-19 vaccine differed by race and ethnicity (Figure 1). In the baseline survey, 28% (8/28) of Black and 21% (6/29) of other non-White respondents reported they were unwilling or very unwilling to receive the COVID-19 vaccine, compared to only 13% (34/268) of White respondents. Among Hispanic/Latinx individuals, 40% (12/40) reported they were unwilling or very unwilling to receive the COVID-19 vaccine compared to 11% (39/300) of non-Hispanic/Latinx respondents.

**Figure 2:**
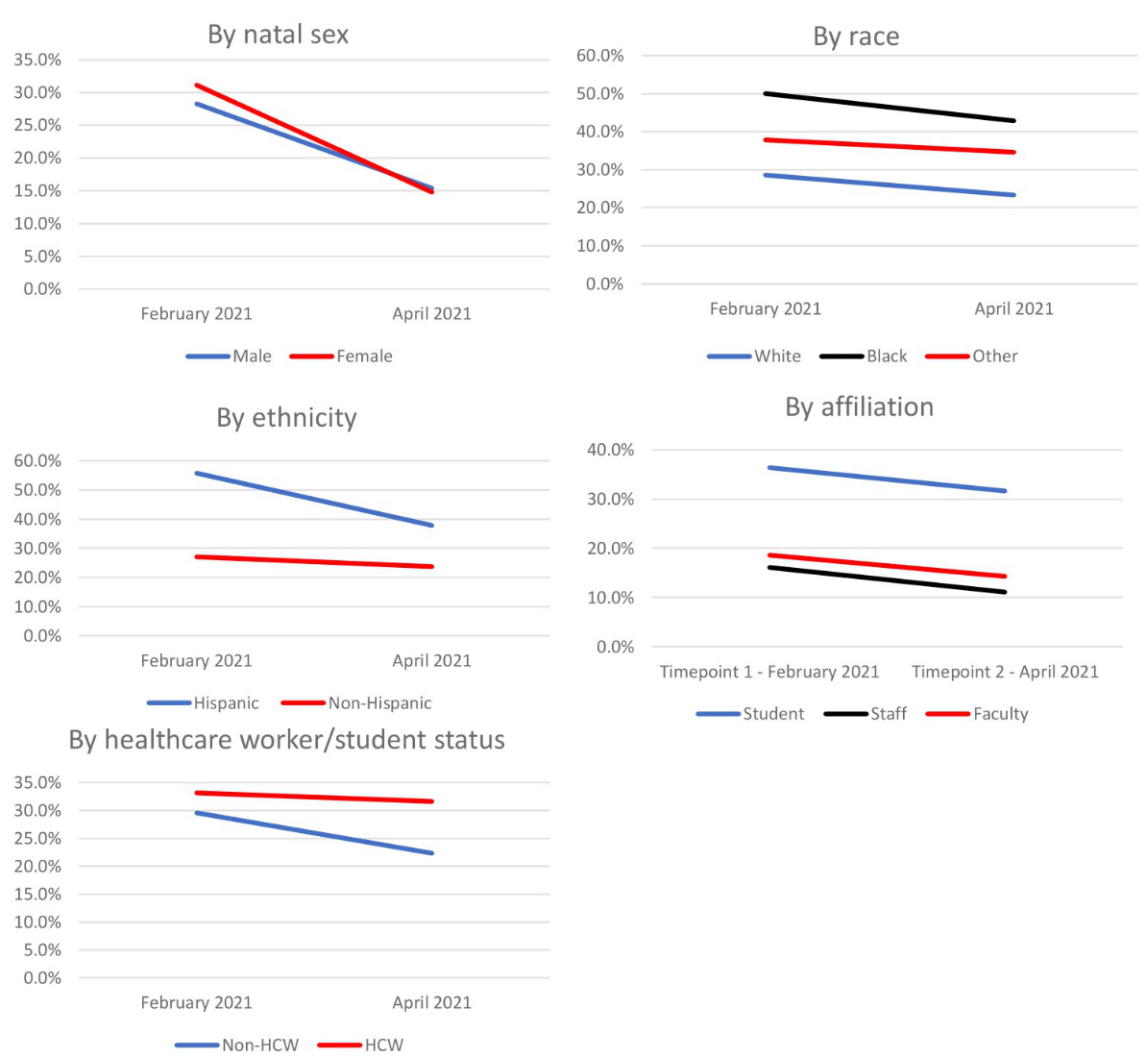
Percentage of participants who believed the COVID-19 vaccine was approved too quickly. This question was directed to the entire cohort (N=451 for time point 1 and N=365 for time point 2). HCW: health care worker.

Participants were also asked about their perceptions of the speed with which the COVID-19 vaccines were granted Emergency Use Authorization by the US Food and Drug Administration. In the baseline survey, 30.4% (137/451) of participants believed the COVID-19 vaccine was approved too quickly (Figure 2, Table S2 in Multimedia Appendix 1). These perceptions differed across racial and ethnic groups: 29% (104/363) of White, 50.0% (15/30) of Black, 37.8% (14/37) of other non-White respondents, and 53.8% (29/52) of Hispanic/Latinx participants believed the COVID-19 vaccine was approved too quickly. In the April survey, 25.1% (95/365) of respondents believed the COVID-19 vaccines were approved too quickly. By race and ethnicity, about 23.3% (74/318) of White, 43% (9/21) of Black, 34.6% (9/26) of other non-White, and 37.8% (14/37) of non-Hispanic/Latinx participants believed the COVID-19 vaccine was approved too quickly in the April survey.

At the final study assessment during late April 2021, about two-thirds (305/454) of all participants reported having received the COVID-19 vaccine (Table 1); this was similar to the Massachusetts statewide vaccination rate at the time [20]. However, in this study, 60.3% (186/308) of student participants reported having received the COVID-19 vaccine; this was much higher than the Massachusetts age-specific vaccination rate among 20- to 29-year-olds at that time, which was approximately 46% [20]. Participants who were natal sex women were about 10% more likely to self-report having received the COVID-19 vaccine compared to natal sex men (Table 1). Black and other non-White participants were 25% less likely to report vaccination compared to White participants. Overall Massachusetts data from the same point in time revealed a similar but weaker relationship between race and vaccination status [20]. However, there was not significant evidence of a relationship between self-reported vaccination uptake among Hispanic/Latinx participants when compared to non-Hispanic/Latinx participants. Taken together, these data indicate disparities in vaccine uptake among Black participants.

Other demographic variables were also found to be significantly associated with self-reported vaccination rate. Staff and faculty were nearly 50% more likely to report vaccination compared to students (Table 1). Of note, the average age among students was 21.5 years, while the average ages among faculty and staff were 50.7 years and 48.2 years, respectively; as such, faculty and staff had a longer window of time during which they could have received the COVID-19 vaccine by the time the survey was conducted due to the age-bracketed rollout in Massachusetts.

Further, we analyzed the relationship between work and/or study in health care settings and self-reported vaccination. Participants who identified as students or workers in health care settings were nearly 30% more likely to report COVID-19 vaccination compared to those who do not work or study in these settings (Table 1). Those who worked/studied in health care settings were given earlier eligibility to receive the COVID-19 vaccine; however, all residents of Massachusetts had been eligible to receive the vaccine since April 19, 2021, and the time of final survey collection was in late April. At the time, there were no workplace requirements for vaccination in the state of Massachusetts.

**Figure 3:**
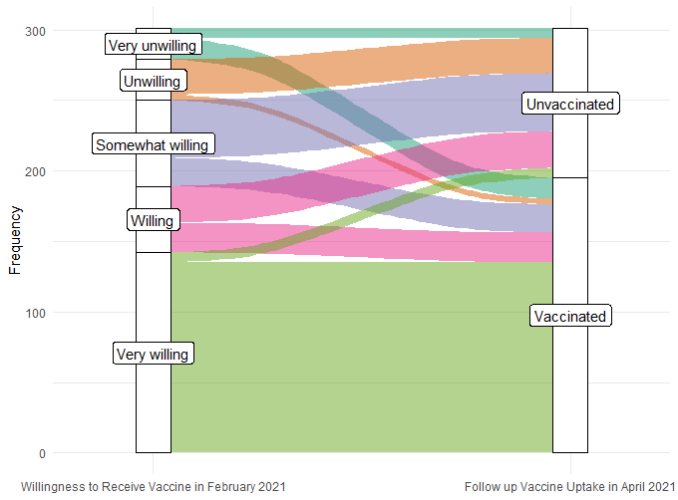
COVID-19 vaccine willingness in February 2021, and follow-up vaccine uptake in April 2021. Participants’ self-reported willingness to receive the COVID-19 vaccine in February 2021 and self-reported vaccine status as of late April 2021. Fisher exact *t* test revealed a significant (*P*<.001) difference in the distribution of baseline vaccine willingness among those unvaccinated versus vaccinated in April 2021.

We examined the relationship between vaccine intention measured at baseline and vaccine uptake in the final survey. Those who responded at baseline that they were likely or very likely to receive the COVID-19 vaccine were significantly more likely to have received the vaccine by the final time point compared to those who said they were unlikely or very unlikely to receive the COVID-19 vaccine, which may indicate that vaccine perception and intentions were formed early in the vaccine rollout process (Figure 3).

Respondents who reported being unwilling to receive the COVID-19 vaccine at the final time point were asked to indicate possible reasons for being hesitant (Table 2). Although the plurality of respondents indicated “None of the above,” the most commonly selected prespecified reason was that the vaccine approval process was rushed. These results were consistent with results regarding questions about whether the vaccine approval timeline was rushed, suggesting that participants’ skepticism of the speed with which the COVID-19 vaccines were approved might have influenced their hesitancy in uptake of the vaccine.

**Table 2:** Self-reported reasons for hesitancy regarding the COVID-19 vaccine among those in the Curry College Community who were unvaccinated as of April 2021 (N=145; respondents could indicate more than one response)^a^.

Reason(s) for hesitancy	The vaccine will give me COVID-19, n (%)	The vaccine affects fertility, n (%)	Natural infection will protect me, n (%)	The approval process was rushed, n (%)	The vaccine is new technology, n (%)	Don’t perceive risk for COVID-19, n (%)	None of the above, n (%)
Responses to this question (N=145)	4 (2.8)	22 (15.2)	5 (11.1)	34 (23.4)	18 (12.4)	9 (6.2)	53 (36.6)

^a^This question was directed at those who reported they were unvaccinated as well as those who said they were unlikely or very unlikely to receive the COVID-19 vaccine.

## Discussion

### Principal Findings

In a cohort of university students, faculty, and staff at a small liberal arts college near Boston, Massachusetts, over two-thirds of participants reported receiving the COVID-19 vaccine by late April 2021. At this time, the COVID-19 vaccine was approved for Massachusetts adults, and the Commonwealth had entered Phase IV of reopening, with some limited indoor gathering and dining [21]. In the previous 2 weeks, there were 53 new cases in Milton, Massachusetts, and 9740 across the state of Massachusetts, down from a high of 72,000 statewide cases in January [20]. About two-thirds of those who were unvaccinated at the completion of the study reported that they would be willing to receive the COVID-19 vaccine once it was available. These findings are reassuring as they indicate the potential to reach a high population vaccination rate that may surpass the community herd immunity threshold [22]. Moreover, these findings indicate that young adult and college student populations may be more amenable to receiving the COVID-19 vaccine than vaccines for other respiratory pathogens, such as the annual influenza vaccine, when compared to previous reports [15]. This could be due for several reasons, including the relative attention placed on the ongoing COVID-19 pandemic compared to other respiratory pathogen epidemics and the extent to which the COVID-19 pandemic has disrupted daily life.

We found significant differences in self-reported vaccination rates by race. The rates of self-reported vaccination among those who were White were significantly higher than the rates among those who were non-White. These results are consistent with statewide data from the Massachusetts Department of Public Health regarding disparities across racial and minority groups, indicating that racial and ethnic minorities, even among college student populations, are comparatively hesitant to receive the COVID-19 vaccine [20]. Thus, although this college population as a whole may achieve population herd immunity, there may be minority subpopulations still at risk for viral spread. This is particularly concerning, as numerous studies have noted racial disparities in clinical outcomes among those infected with COVID-19: not only are these individuals more at risk of becoming infected with COVID-19, but they are also more likely to have adverse outcomes compared to White individuals [23].

Furthermore, among those who were unvaccinated, we found significant differences in perceptions surrounding the willingness to receive the COVID-19 vaccine and perceptions of the vaccine across natal sex and race strata. We observed that Black and other non-White individuals were significantly more likely to say they were unwilling to receive the COVID-19 vaccine when available compared to their White counterparts. We found that Black and other non-White participants were significantly more likely to say that the COVID-19 vaccine was approved too quickly, and many cited this as being a reason they were hesitant to receive the COVID-19 vaccine. These disparities persisted in our cohort from the baseline survey to the final survey, despite increasing national attention focusing on disparities in vaccine uptake.

Lastly, we examined the relationship between intention to be vaccinated and self-reported receipt of vaccine. We found an association between baseline vaccine intention (ie, intent to receive the vaccine or lack thereof) and self-reported vaccination, which may indicate that perceptions and personal intent surrounding the vaccine formed early on in the vaccine rollout process influenced the decision to be vaccinated. This result may inform future public health messaging campaigns aimed at minority community outreach.

The disparities in vaccine uptake and vaccine hesitancy reported in many settings in the United States [24-26] were also observed among these college students, staff, and faculty. This highlights the need for targeted approaches and interventions among racial and ethnic minorities to increase vaccine uptake on college campuses. Particularly with the prevalent Delta variant’s increased transmissibility, college administrators and public health officials must understand racial differences in vaccine perceptions to help prevent the spread of COVID-19 in their communities.

### Limitations

Our data have important limitations. First, the data were collected using a convenience sample of Curry College community members and are therefore subject to selection bias. Recruitment was completed by mass email to all students, faculty, and staff that would be on campus at Curry College at least part-time in the Spring 2021 semester, as well as in-person at the Student Health SARS-CoV-2 polymerase chain reaction testing site. In total, about 14% of the total student population participated. Of student participants, nearly three-quarters reported living on campus compared to about 40% of the overall student population. It is possible that those who chose to complete some in-person instruction/work were less risk-averse and/or were self-assessed to be at less risk of severe COVID-19 compared to those who chose entirely virtual instruction/work. This difference in risk aversion may or may not correlate with other beliefs surrounding COVID-19, including perceptions and behaviors regarding the vaccine. However, due to the nature of this study and the necessity of on-campus presence to collect venipuncture samples, inclusion of fully remote students and workers was not possible. Moreover, the sampled population of students was more likely to be White and non-Hispanic compared to the overall Curry College student population. There are many possible reasons for fewer non-White students electing to participate. This may indicate that non-White students opted to pursue virtual instruction at a different rate than White students. Non-White students may also have been less likely to self-enroll in a health-related study due to distrust of the medical/public health system due to historic mistreatment and institutionalized racism [27,28].

Additionally, the Curry College campus population from which the cohort of participants was drawn is itself largely non-Hispanic White. Furthermore, Curry College is a private liberal arts school located in Milton, Massachusetts, a suburb of Boston, with a median income of $133,718 [29]. As such, this community has a higher education level and higher income compared to the state as a whole, which may limit the transferability of these data. Indeed, at the time of the conclusion of the study, Milton had a higher local vaccination rate (57%) compared to the overall Massachusetts average (52.3%) [15], which may indicate that this community’s perceptions surrounding COVID-19 vaccine uptake differ from elsewhere in the state. In addition, our sample also had a substantial proportion of health care workers, even among students, many of whom were vaccinated once vaccines were made available. Therefore, our cohort may not be representative of all university students nationwide or Massachusetts as a whole.

### Conclusion

In a population of students, faculty, and staff at a New England residential college, a large majority were either vaccinated or willing to be vaccinated against COVID-19 by late April 2021. However, Black and other non-White racial groups were significantly less likely to be vaccinated or willing to be vaccinated. Perceptions of the COVID-19 vaccine in February 2021 predicted vaccination uptake by April, suggesting that views of the vaccine formed soon after it was approved shaped vaccine adoption behaviors. In this unique cohort, we observed lower vaccination willingness and uptake among racial/ethnic minority populations, similar to studies of other US populations. Additional research and programmatic activities are needed to understand reasons for vaccine hesitancy and to overcome hesitancy to work toward equitable vaccine coverage.

## Appendix

Multimedia Appendix 1 Supplemental Tables: Sampling Fractions and Survey Responses of Curry College community members, Milton, Massachusetts, in February and April 2021.

## Abbreviations

EUA: Emergency Use Authorization
HCW: health care worker/student
