# Supplementary material for: COVID-19 Vaccine Perceptions, Intentions, and Uptake Among Young Adults in the United States: Prospective College-Based Cohort Study
Source: JMIR Public Health Surveill. 2021 Dec 15;7(12):e33739. doi: 10.2196/33739 (PMC8675562; doi:10.2196/33739)
Supplement: Multimedia Appendix 1 [file publichealth_v7i12e33739_app1.docx]

**Table S1: Respondent Demographics by Timepoint and Overall Curry College Student Demographics**

|  | **All Participants** | |  | **All Participants** | |  | **All Curry Students** | |
| --- | --- | --- | --- | --- | --- | --- | --- | --- |
|  | **February 2021 (N=454)** | |  | **April 2021(N=328)** | |  | **20-21 AY (N=2,206)** | |
|  | **n** | **(%)** |  | **n** | **(%)** |  | **n** | **(%)*** |
| **Natal Sex** |  |  |  |  |  |  |  |  |
| **Male** | 121 | 26.70% |  | 68 | 20.7% |  | 842 | 38.10% |
| **Female** | 333 | 73.30% |  | 261 | 79.3% |  | 1360 | 61.70% |
| **Race** |  |  |  |  |  |  |  |  |
| **White** | 366 | 80.60% |  | 282 | 89.2% |  | 1415 | 64.14% |
| **Black** | 33 | 7.30% |  | 20 | 6.3% |  | 314 | 14.23% |
| **Other** | 34 | 7.50% |  | 14 | 4.4% |  | 319 | 14.46% |
| **Ethnicity** |  |  |  |  |  |  |  |  |
| **Not Hispanic/Latinx** | 402 | 88.50% |  | 301 | 91.8% |  | 1871 | 84.81% |
| **Hispanic/Latinx** | 51 | 11.20% |  | 27 | 8.2% |  | 177 | 8.02% |
| **Affiliation** |  |  |  |  |  |  |  |  |
| **Student** | 308 | 67.80% |  | 205 | 62.5% |  | 2206 | 100% |
| **Staff** | 86 | 18.90% |  | 69 | 21.0% |  | --- | --- |
| **Faculty** | 56 | 12.30% |  | 54 | 16.5% |  | --- | --- |

**Table S2: Perceptions surrounding the COVID-19 vaccine among Curry College community members, Milton Massachusetts, at baseline time point, February 2021.**

|  |  | **N (%)** | | | | |
| --- | --- | --- | --- | --- | --- | --- |
| **Among those who were unvaccinated at final time point** | ***Likelihood of getting vaccinated*** | **Very Unlikely**  **N (%)** | **Unlikely N (%)** | **Somewhat Likely**  **N (%)** | **Likely**  **N (%)** | **Very Likely**  **N (%)** |
| **Responses to this Question: N=341** | | 22 (6.5) | 29 (8.5) | 61 (17.9) | 47 (13.8) | 182 (53.4) |
| **Natal Sex** | **Female (234)** | 15 (6.4) | 18 (7.7) | 46 (19.7) | 33 (14.1) | 122 (52.1) |
|  | **Male (107)** | 7 (6.5) | 11 (37.9) | 15 (14.0) | 14 (13.1) | 60 (56.1) |
| **Race** | **White (268)** | 20 (7.5) | 14 (5.2) | 42 (15.7) | 43 (16.0) | 149 (86.1) |
|  | **Black (28)** | 1 (3.6) | 7 (25.0) | 10 (35.7) | 2 (7.1) | 8 (28.6) |
|  | **Other (includes multiracial) (29)** | 0 (0.0) | 6 (20.7) | 7 (24.1) | 0 (0.0) | 16 (55.2) |
| **Ethnicity** | **Non-Hispanic (300)** | 20 (6.7) | 19 (6.3) | 49 (16.3) | 44 (14.7) | 168 (56) |
|  | **Hispanic (40)** | 2 (5.0) | 10 (25.0) | 12 (30) | 3 (3.5) | 13 (32.5) |
| **Affiliation** | **Student (215)** | 8 (3.7) | 27 (12.6) | 52 (24.2) | 37 (17.2) | 91 (42.3) |
|  | **Faculty (47)** | 0 (0.0) | 0 (0.0) | 3 (6.4) | 4 (8.5) | 40 (85.1) |
|  | **Staff (77)** | 14 (18.2) | 1 (1.3) | 7 (9.09) | 6 (7.8) | 49 (63.6) |
| **HCW** | **Non-HCW (80)** | 8 (10.0) | 6 (7.5) | 13 (16.6) | 17 (21.3) | 36 (45.0) |
|  | **HCW (25)** | 0 (0.0) | 2 (8.0) | 8 (32.0) | 5 (20.0) | 10 (40.0) |
|  | ***Vaccines are safe.*** | **Strongly Disagree** | **Disagree** | **Neither Agree nor Disagree** | **Agree** | **Strongly Agree** |
| **Responses to this Question:N=51** | | **2 (3.9)** | **3 (5.9)** | **14 (27.6)** | **18 (35.3)** | **14 (27.5)** |
|  |  |  |  |  |  |  |
| **Responses asked among total cohort** | ***Vaccines are effective.*** | **Strongly Disagree** | **Disagree** | **Neither Agree nor Disagree** | **Agree** | **Strongly Agree** |
| **Responses to this Question: N=452** | | **4 (0.88)** | **2 (0.44)** | **39 (8.63)** | **167 (36.95)** | **240 (53.10)** |
| **Natal Sex** | **Female (332)** | 4 (1.20) | 1 (0.30) | 30 (9.04) | 122 (36.75) | 175 (52.71) |
|  | **Male (120)** | 0 (0.00) | 1 (0.83) | 9 (7.50) | 45 (37.50) | 65 (54.17) |
| **Race** | **White (364)** | 3 (0.82) | 1 (0.27) | 23 (6.32) | 128 (35.16) | 209 (57.42) |
|  | **Black (30)** | 0 (0.00) | 1 (3.33) | 7 (23.33) | 14 (46.67) | 8 (26.67) |
|  | **Other (incl multiracial) (37)** | 0 (0.00) | 0 (0.00) | 7 (18.92) | 16 (43.24) | 14 (37.84) |
| **Ethnicity** | **Non-Hispanic (400)** | 3 (0.75) | 1 (0.25) | 25 (6.25) | 149 (37.25) | 222 (55.50 |
|  | **Hispanic (52)** | 1 (1.92) | 1 (1.92) | 14 (26.92) | 18 (34.62) | 18 (34.62) |
| **Affiliation** | **Student (306)** | 1 (0.33) | 2 (0.65) | 31 (10.13) | 117 (38.24) | 155 (50.65) |
|  | **Faculty (56)** | 2 (3.57) | 0 (0.00) | 0 (0.00) | 13 (23.21) | 41 (73.21) |
|  | **Staff (86)** | 1 (1.16) | 0 (0.00) | 6 (6.98) | 35 (40.70) | 44 (51.16) |
| **HCW** | **Non-HCW (312)** | 4 (1.28) | 1 (0.32) | 31 (9.94) | 120 (38.46) | 156 (50.00) |
|  | **HCW (140)** | 0 (0.00) | 1 (0.71) | 8 (5.71) | 47 (33.57) | 84 (60.00) |
| **Responses among total Cohort** | ***Timing of vaccine approval*** | **Approved much too soon (%)** | **Approved too soon (%)** | **approved at just the right time (%)** | **approved too late (%)** | **approved much too late (%)** |
| **Responses to this Question: N=451** | | 20 (4.43) | 117 (25.94) | 269 (59.65) | 40 (8.87) | 5 (1.11) |
| **Natal Sex** | **Female (331)** | 16 (4.83) | 87 (26.28) | 196 59.21) | 28 (8.46) | 4 (4.83) |
|  | **Male (120)** | 4 (3.33 | 30 (25.00) | 73 (60.83) | 12 (10.00) | 1 (0.83) |
| **Race** | **White (363)** | 15 (4.13) | 89 (24.52) | 227 (62.53) | 27 (7.44) | 5 (1.38) |
|  | **Black (30)** | 2 (6.67) | 13 (43.33) | 13 (43.33) | 2 (6.67) | 0 (0.00) |
|  | **Other (incl multiracial) (37)** | 2 (5.41) | 12 (32.43) | 14 (37.84) | 9 (24.32) | 0 (0.00) |
| **Ethnicity** | **Non-Hispanic (399)** | 13 (3.26) | 95 (23.81) | 253 (63.41) | 34 (8.52) | 4 (1.00) |
|  | **Hispanic (52)** | 7 (13.46) | 22 (42.31) | 16 (30.77) | 6 (11.54) | 1 (1.92) |
| **Affiliation** | **Student (305)** | 18 (5.90) | 93 (30.49) | 161 (52.79) | 30 (9.84) | 3 (0.98( |
|  | **Faculty (56)** | 1 (1.79 | 8 (14.29) | 41 (73.21) | 5 (8.93) | 1 (1.79) |
|  | **Staff (86)** | 1 (1.16) | 15 (17.44) | 64 (74.42) | 5 (5.81) | 1 (1.16) |
| **HCW** | **Non-HCW (311)** | 9 (2.89) | 83 (26.69) | 188 (60.45) | 28 (9.00) | 3 (0.96) |
|  | **HCW (140)** | 11 (7.86) | 34 (24.29) | 81 (57.86) | 12 (8.57( | 2 (1.43) |

**Table S3: Perceptions surrounding the COVID-19 vaccine among Curry College community members, Milton Massachusetts, at final timepoint, April 2021.:**

|  |  | **n(%)** | | | | |
| --- | --- | --- | --- | --- | --- | --- |
| **Among those who were unvaccinated at final time point** | ***Likelihood of getting vaccinated*** | **Very Unlikely (%)** | **Unlikely (%)** | **Unsure (%)** | **Likely (%)** | **Very Likely (%)** |
| **Responses to this Question: N=105** | | 8 (7.62) | 8 (7.62) | 21 (20.00) | 22 (20.95) | 46 (43.81) |
| **Natal Sex** | **Female (290)** | 2 (7.41) | 2 (7.41) | 5 (18.52) | 11 (40.74) | 7 (25.93) |
|  | **Male (78)** | 6 (7.69) | 6 (7.69) | 16 (20.51) | 11 (14.10) | 39 (50.00) |
| **Race** | **White (83)** | 5 (6.02) | 6 (7.23) | 12 (14.46) | 19 (22.89) | 41 (49.40) |
|  | **Black (10)** | 1 (10.00) | 1 (10.00) | 6 (60.00) | 1 (10.00) | 1 (10.00) |
|  | **Other (incl multiracial) (10)** | 1 (10.00) | 1 (10.00) | 3 (30.00) | 1 (10.00) | 4 (40.00) |
| **Ethnicity** | **Non-Hispanic (93)** | 5 (5.38) | 6 (6.45) | 17 (18.28) | 21 (22.58) | 44 (47.31) |
|  | **Hispanic (12)** | 3 (25.00) | 2 (16.67) | 4 (33.33) | 1 (8.33) | 2 (16.67) |
| **Affiliation** | **Student (87)** | 5 (5.75) | 7 (8.05) | 20 (22.99) | 18 (20.69) | 37 (42.53) |
|  | **Faculty (7)** | 2 (28.57) | 0 (0.00) | 0 (0.00) | 2 (28.57) | 3 (42.86) |
|  | **Staff (11)** | 1 (9.09) | 1 (9.09) | 1 (9.09) | 2 (18.18) | 6 (54.55) |
| **HCW** | **Non-HCW (80)** | 8 (10.00) | 6 (7.50) | 13 (16.25) | 17 (21.25) | 36 (45.00) |
|  | **HCW (25)** | 0 (0.00) | 2 (8.00) | 8 (32.00) | 5 (20.00) | 10 (40.00) |
| **Responses among total cohort** | ***Vaccines are safe*** | **Strongly Disagree (%)** | **Disagree (%)** | **Neither Agree nor Disagree (%)** | **Agree (%)** | **Strongly Agree (%)** |
| **Responses to this Question: N=379** | | 2 (0.53) | 4 (1.06) | 36 (9.50) | 143 (37.73) | 196 (51.72) |
| **Natal Sex** | **Female (290)** | 2 (0.69) | 2 (0.69) | 25 (8.62) | 109 (37.59) | 152 (52.41) |
|  | **Male (89)** | 0 (0.00 | 0 (0.00) | 11 (12.36) | 34 (38.20) | 44 (49.44) |
| **Race** | **White (318)** | 1 (0.31) | 2 (0.63) | 26 (8.18) | 117 (36.79) | 172 (54.09) |
|  | **Black (21)** | 0 (0.00) | 0 (0.00) | 7 (33.33) | 9 (42.86) | 5 (23.81) |
|  | **Other (incl multiracial) (26)** | 0 (0.00) | 0 (0.00) | 3 (11.54) | 11 (42.31) | 12 (46.15) |
| **Ethnicity** | **Non-Hispanic (342)** | 1 (0.29) | 2 (0.58) | 30 (8.77) | 125 (36.55) | 184 (53.80) |
|  | **Hispanic (37)** | 1 (2.70) | 0 (0.00) | 6 (16.22) | 18 (48.65) | 12 (32.43) |
| **Affiliation** | **Student (87)** | 1 (0.40) | 1 (0.40) | 33 (13.36) | 91 (36.84) | 121 (48.99) |
|  | **Faculty (7)** | 0 (0.00) | 0 (0.00) | 1 (1.85) | 15 (27.78) | 38 (70.37) |
|  | **Staff (11)** | 1 (1.30) | 1 (1.30) | 2 (2.60) | 36 (46.75) | 37 (48.05) |
| **HCW** | **Non-HCW (80)** | 2 (0.75) | 2 (0.75) | 29 (10.94) | 100 (37.74) | 132 (49.81) |
|  | **HCW (25)** | 0 (0.00) | 0 (0.00) | 7 (6.14) | 43 (37.72) | 64 (56.14) |
| **Responses among total cohort** | ***Vaccines are effective*** | **Strongly Disagree (%)** | **Disagree (%)** | **Neither Agree nor Disagree (%)** | **Agree (%)** | **Strongly Agree (%)** |
| **Responses to this Question: N=379** | | 1 (0.26) | 4 (1.06) | 27 (7.12) | 159 (41.95) | 188 (49.60) |
| **Natal Sex** | **Female (290)** | 0 (0.00) | 0 (0.00) | 6 (6.74) | 43 (48.31) | 40 (44.94) |
|  | **Male (89)** | 1 (0.34) | 4 (1.38) | 21 (7.24) | 116 (40.00) | 148 (51.03) |
| **Race** | **White (318)** | 1 (0.31) | 3 (0.94) | 21 (6.60) | 127 (39.94) | 166 (52.20) |
|  | **Black (21)** | 0 (0.00) | 0 (0.00) | 4 (19.05) | 13 (61.90) | 4 (19.05) |
|  | **Other (incl multiracial) (26)** | 0 (0.00) | 0 (0.00) | 2 (7.69) | 13 (50.00) | 11 (42.31) |
| **Ethnicity** | **Non-Hispanic (342)** | 1 (0.29) | 3 (0.88) | 22 (6.43) | 136 (39.77) | 180 (52.63) |
|  | **Hispanic (37)** | 0 (0.00) | 1 (2.70) | 5 (13.51) | 23 (62.16) | 8 (21.62) |
| **Affiliation** | **Student (247)** | 1 (0.40) | 2 (0.81) | 22 (891) | 104 (42.11) | 118 (47.77) |
|  | **Faculty (54)** | 0 (0.00) | 0 (0.00) | 2 (3.70) | 18 (33.33) | 34 (62.96) |
|  | **Staff (77)** | 0 (0.00) | 2 (2.60) | 3 (3.90) | 36 (46.75) | 36 (46.75) |
| **HCW** | **Non-HCW (265)** | 1 (0.38) | 4 (1.51) | 21 (7.92) | 112 (42.26) | 127 (47.92) |
|  | **HCW (114)** | 0 (0.00) | 0 (0.00) | 6 (5.26) | 47 (41.23) | 61 (53.51) |
| **Responses among total cohort** | ***Timing of vaccine approval*** | **Approved too late (%)** | **Approved at the right time (%)** | **Approved too Quickly (%)** | **I don't know (%)** |  |
| **Responses to this Question: N=365** | | 15 (3.96) | 217 (57.26) | 95 (25.07) | 52 (13.72) |  |
| **Natal Sex** | **Female (290)** | 4 (4.49) | 55 (61.80) | 17 (19.10) | 13 (14.61) |  |
|  | **Male (89)** | 11 (3.70) | 162 (55.86) | 78 (26.90) | 39 (13.45) |  |
| **Race** | **White (318)** | 12 (3.77) | 189 (59.43) | 74 (23.27) | 43 (13.52) |  |
|  | **Black (21)** | 0 (0.00) | 8 (38.10) | 9 (42.86) | 4 (19.05) |  |
|  | **Other (incl multiracial) (26)** | 2 (7.69) | 11 (42.31) | 9 (34.62) | 4 (15.38) |  |
| **Ethnicity** | **Non-Hispanic (342)** | 13 (3.80) | 203 (59.36) | 81 (23.68) | 45 (13.16) |  |
|  | **Hispanic (37)** | 2 (5.41) | 14 (37.84) | 14 (37.84) | 7 (18.92) |  |
| **Affiliation** | **Student (247)** | 8 (3.24) | 126 (51.01) | 78 (31.58) | 35 (14.17) |  |
|  | **Faculty (54)** | 3 (5.56) | 38 (70.37) | 6 (11.11) | 7 (12.96) |  |
|  | **Staff (77)** | 4 (5.19) | 52 (67.53) | 11 (14.29) | 10 (12.99) |  |
| **Healthcare Worker/Student (HCW)** | **Non-HCW (265)** | 12 (4.53) | 153 (57.74) | 59 (22.26) | 41 (15.47) |  |
|  | **HCW (114)** | 3 (2.63) | 64 (56.14) | 36 (31.58) | 11 (9.65) |  |
